# On to Nashville

**Published:** 1897-08

**Authors:** 


### U. S. V. M. A.

### ON TO NASHVILLE!

The fourth annual meeting of the Association of Veterinary Faculties of North America will be held in connection with U. S. V. M. A., at Nashville, Tenn., Wednesday, September 8, 1897. The following papers will be read: "Uniform Courses of Instruction," Dr. John W. Adams, University of Pennsylvania; "Minimum Standard of Entrance Examinations," Drs. D. E. Salmon, Columbian University, and James Law, Cornell University; "Uniformity of State Regulations Governing the Practice of Veterinary Medicine," Dr. W. Horace Hoskins, Philadelphia. The following are the officers of the association: President, Dr. Leonard Pearson; Secretary and Treasurer, Dr. H. D. Gill, 154 E. Fifty-seventh Street, New York City: Executive Committee, James Law, M. Stalker, D. McEachran, D. E. Salmon, and J. L. Robertson.

Discussion of tuberculosis, including the following topics: "Results Attained and Attainable by State Control;" "Value, Defects, and Difficulties of Municipal Control;" "What Should be Done with Tuberculous Cattle?" "What Use Should be Made of the Milk from Tuberculous Cows?" "What Use Should be Made of the Flesh of Tuberculous Animals?"

Chicago veterinarians are favored by excursion rates to Nashville and return for the sum of $10.80.

The following papers will be read at the convention: "The Disposition of the Flesh of Tuberculous Animals," and "A Review of the Field of Veterinary Science," Dr. Leonard Pearson: "The Action, Experimentally, of Tuberculin on Healthy Animals," Dr. T. D. Hinebauch; "Our Milk-supply," Dr. Charles Ellis; "Infectious Mammitis in Cows," Dr. E. A. A. Grange; "Malignant Catarrh of the Ox," Dr. A. Youngberg; "Texas-fever Investigation," Dr. J. W. Connoway; "Radical Operation for Contracted Hoof," Dr. C. C. Lyford; "Some Diseases of the Horse Peculiar to the Mountain Region," Dr. M. E. Knowles; "The Veterinary Field in the South," Dr. W. H. Dalrymple; "The Necessity of Veterinary Instruction in Medical Colleges," Dr. E. P. Niles; "Osteoporosis," Dr. C. A. Cary. Drs. Wm. Dougherty, S. J. J. Harger, and others will also read papers, the subjects of which, however, are not yet announced.

The programme of entertainment of the United States Veterinary Medical Association, September 7th, 8th, and 9th, will be as follows: Headquarters, Tulane Hotel. Address of Welcome, by Hon. John J. McCann; response by President of the Association; business of the Association; ladies taken in charge by local committee, visiting Centennial in a body.

*Second day, morning.* Ladies visit Capitol, Phillips & Buttoroff Manufacturing Co., and other places of interest. *Afternoon.* Both ladies and gentlemen congregate at Union Depot for a trip to Belle Meade; returning at night, stop at Exposition.

**Where will the meeting of 1898 convene?**

*Third day.*—Go-as-you-please for gentlemen; ladies visit Exposition, and take trolley ride around the city; banquet at Tulane.

The health officers of Nashville and of Davidson County report an excellent sanitary condition of all the Exposition grounds and buildings, the general freedom from disease of all kinds of the city of Nashville during June. This augurs well for our convention, for September is said to be a specially pleasant month of the year in that territory.

It is the veterinarian who never attends association meetings that say they do not pay, and they cannot afford the time or expense.

Veterinarians in the Eastern States are offered an extremely pleasant trip to Nashville, and the following attractive rates via M. & M. T. Co., Boston to Nashville and return. Tickets on sale Tuesdays and Thursdays, good fourteen days, in addition to date of sale, $32.65; tickets on sale daily, good twenty-four days, in addition to date of sale, $38.05. These rates include meals and state-room accommodations on M. & M. T. Co.'s steamer. Upper-deck state-room berths are $1.50 extra in each direction. The steamer leaving Boston Thursday, September 2d, at 2 P.M. is due at Norfolk 4 A.M., September 4th. Train via Southern Railway leaves Norfolk 9.25 A.M., due in Nashville 1.35 P.M., Sunday, September 5th, the only line running through car-service from Norfolk to Nashville, passing through the most beautiful scenery of Western North Carolina, the "Land of the Sky," thence Knoxville and Chattanooga to Nashville.

**Nashville will point the way for '98 and '99.**

On page 466 of the July JOURNAL, referring to those wishing to attend Nashville from Philadelphia, Tuesday, September 3d, should read Thursday.

$32 65 will take you from Boston to Nashville and return; this includes meals and state-room on steamer. New Yorkers may obtain a ten days' excursion rate for Nashville on Tuesdays and Thursdays for $25.30. Members from Washington may obtain a ten-day rate of $15.30 from Washington to Nashville and return.

Rates via Pennsylvania Railroad to Nashville: For a season excursion rate from New York via Pittsburg, $38.50; via Washington and C. & O., $35.50; from Philadelphia, $34.75 and $33.25; from Baltimore, $30.55, and the same via Washington.

Dr. A. W. Clement, of Baltimore, Maryland, will present the subject of "Rabies" at Nashville, and will view it from the standpoint of State medicine and the part to be taken in its control and suppression.

Dr. George Kinnell, of Pittsfield, Mass., will read a paper on "Toxalbumins and Antitoxins," at Nashville.

All veterinarians in New York State, New Jersey, Eastern and Central Pennsylvania, Delaware, Maryland, District of Columbia, and Virginia will find the special ten-day excursion rates of the Southern Railway Company a very favorable way for reaching Nashville.

The "Land of the Sky" from Asheville to Chattanooga will be a revelation of beauty to those who have never passed through that wonderfully attractive and interesting country.

The Washington and Southwestern vestibuled limited, leaving New York at 4.25 P.M., Philadelphia 6.55 P.M, Baltimore 9.20 P.M., Washington 10.43 P.M., on Tuesday and Thursday passes through the "Land of the Sky" by daylight.

Drs. D. E. Salmon and E. P. Niles, M. H. Reynolds, and John M. Parker will specially discuss the tuberculosis question. This insures a keen interest in this all-important topic.

It looks like a great outpouring of the profession at Nashville. A list of those who have indicated their intention of being present includes Drs. S. B. Nelson, Pullman, Wash.; M. E. Knowles, Butte, Mont.; T. S. Hinebauch, Fargo, Dak.; M. R. Trumbower, Denver, Col.; A. S. Wheeler, Biltmore, N. C.; W. H. Dalrymple, Baton Rouge, La.; J. F. Winchester, Lawrence, Mass, and Dr. J. C. Norton, of Arizona, and many others. Large delegations will attend from Minnesota, Indiana, Illinois, and Missouri.

New York State Veterinary College will be represented by Professors Law and Williams.

Remember that all the main passenger lines have reduced rates to Nashville, 20 per cent. off of regular fare for round-trip tickets, daily sale; on special days of each week a still greater reduction for ten, fifteen, and twenty-day limits. This is better than the usual one and one-third rates obtained by the association.

**Many will have their wives at Nashville. How about yours?**

We have received several communications from Western veterinarians advocating a reduction in the annual dues of the U. S. V. M. A. The Executive Committee will probably be asked to consider the advisability of this step.

Among those who are applicants for membership at Nashville, we note the names of Drs. Charles W. Heitzman, of New Orleans, La.; J. C. Robert, Agricultural College, Mississippi; M. Francis, College Station, Texas; G. N. Kinnell, Pittsfield, Mass.; E. M. Ranck, Philadelphia, Pa.; Joseph M. Good, Chattanooga, Tenn.

One of the late announcements of papers for Nashville is one on "Operative Dental Surgery," by Dr. L. A. Merillat, of Chicago, Ill. This is always a topic that gives rise to a variety of opinions, and has been the field of more charlatan work than any branch of veterinary science, and will awaken at Nashville an interesting discussion. Other live subjects are equally in order.

Veterinarians who are not able to purchase tickets direct to Nashville from the point of departure should purchase tickets to the nearest large railroad centre, where the special excursion rates granted by all main lines may be obtained.

**The programme is more than attractive.**

Every member of the association should charge himself with bringing in one new member of the association. There are five hundred good men we could name, all of whom should be members of the U. S. V. M. A. The Secretary will furnish you with application blanks and copies of the constitution and by-laws. When you read this, act on the suggestion made at once. You owe it to the association, and it is your duty to your nearest worthy colleague.

